# Alloreferent and Apparent Seasonal Polyphenism of *Dielis tejensis* with an Updated Key to Nearctic *Dielis* Species (Hymenoptera: Scoliidae) †

**DOI:** 10.3390/insects17030295

**Published:** 2026-03-09

**Authors:** Przemyslaw Szafranski

**Affiliations:** Department of Molelecular and Human Genetics, Baylor College of Medicine, One Baylor Plaza, Houston, TX 77030, USA; pszafran@bcm.edu

**Keywords:** Campsomerini, taxonomy, phenotypic variability, Texas endemism, floral association

## Abstract

Scoliidae are fossorial aculeate wasps (Hymenoptera: Apocrita) whose larvae develop as parasitoids of beetle grubs, mainly from the family Scarabaeidae. Despite their distinctive appearance and economic importance, the taxonomy and biology of Scoliidae are less well understood compared to other Hymenoptera. Recently, a new species of Scoliidae, *Dielis tejensis* Szafr., was described based on about a dozen male specimens from Texas (Nearctic realm). This article reports on mtDNA-verified discovery of the female *D. tejensis*. As in the case of other members of the tribe Campsomerini, *D. tejensis* exhibits strong sexual dimorphism in body structure and color pattern. Females of *D. tejensis* most closely resemble *Dielis plumipes* (Drury) and were previously confused with *D. plumipes fossulana* (Fabr.), with which *D. tejensis* is partially sympatric. *D. tejensis* has more than one generation per year, characterized by a male-biased sex ratio, perhaps resulting from the female immature stages entering a state of aestivation. The multi-generational developmental cycle of *D. tejensis* correlates with the existence of a partial seasonal polyphenism in this species. The data presented also shows a similarity in the distribution ranges of *D. tejensis* and one of its main nectaring plants, *Hymenopappus artemisiifolius* DC (Asteraceae), that may suggest similarity in the environmental requirements of both organisms or specialized trophic relationship between the still-unknown beetle host of the wasp larvae and *H. artemisiifolius* or another plant with a similar range. *D. tejensis* has been included in the accordingly modified key to the Nearctic species of *Dielis* Sauss. & Sichel, which is also presented here.

## Simple Summary

The family Scoliidae consists of parasitoid aculeate wasps with taxonomy that has long been problematic due to frequent interspecific similarities and plesiomorphies which complicate phylogenetic analyses. The biology of Scoliidae is also not yet fully understood, despite their importance for science and the economy. This article describes the female of a recently discovered species of Scoliidae from North America, *Dielis tejensis*. It also presents an updated key for identifying Nearctic representatives of *Dielis*, reports partial seasonal polyphenism in *D. tejensis*, a phenomenon previously unknown in Scoliidae, and hypothesizes about what the similarity of the ranges of *D. tejensis* and one of its major nectaring plants, *Hymenopappus artemisiifolius*, may indicate.

## 1. Introduction

Soliidae are a family of relatively large, fossorial, solitary, stinging wasps (Hymenoptera: Apocrita) with approximately 560 species distributed mainly in the warmer regions of the temperate zone, in the subtropics, and the tropics [1]. According to the majority of contemporary analyses, they, along with Bradynobaenidae, are most closely related to Formicoidea and Apoidea [2,3,4,5,6,7]. Scoliidae develop as idiobiont ectoparasitoids of the final developmental stage of beetle larvae, mainly Scarabaeoidea, which feed underground on roots or in decaying wood and other organic matter [8]. Since some of these beetles are economically important as serious crop pests, several species of Scoliidae have been used as biological agents to control scarab populations [9,10]. Scoliidae also contribute to plant pollination while foraging for nectar, and an array of peptides in their venom have been found to have applications in medicine [11,12]. Recently, Scoliidae have attracted attention due to the discovery in one of their major tribes, Campsomerini, of a mitochondrial gene structure/arrangement previously unknown in animals [13,14,15]. However, despite their unique value for science, economic importance, and striking appearance, the taxonomy of Scoliidae remains unclear in many respects, and information on their biology is scarce (e.g., [16,17,18]). The extant species are currently classified into subfamilies Proscoliinae (with only two species) and Scoliinae, with the latter consisting of tribes Scoliini, Trielidini, and Campsomerini (e.g., [17,19]).

*Dielis* Saussure and Sichel, 1864 (type species: *Dielis plumipes* (Drury), [20]), is the most species-rich genus of Campsomerini in the Nearctic realm, with a distribution likely limited to the Americas [1,21]. It was elevated from a subgenus of *Campsomeris* Lepeletier to generic status by Argaman (1996) [22] and accepted as such in the majority of subsequent publications referring to Nearctic and Neotropical Scoliidae (e.g., [1,17,23]). The genus *Dielis* essentially includes the species groups of *D. plumipes* and *D. pseudonyma* (Schulz) [21], but its composition is not definitively settled. *Dielis pilipes* (Saussure) awaits formal removal from *Dielis* as it not only consistently groups with *Xanthocampsomeris* Bradley in analyses of nuclear ultraconserved DNA sequences [17], but it also does not appear to belong to *Dielis* based on phenotypic characters [21,22,24]. The taxonomic ranks of *Dielis bahamensis* (Bradley) [25] and the subspecies of *D. plumipes* [1] require further investigation. In addition, several Neotropical species are listed in Osten’s catalogue [1] as possibly belonging to *Dielis*. Quite unexpectedly, considering the generally good level of knowledge of North American biodiversity, a new species of *Dielis*, *D. tejensis* Szafranski, 2023, was recently described based on male specimens from the East-central Texas savannas and prairies.

This article reports on the identification of the female *D. tejensis* and partial seasonal polyphenism in this species, discusses new data on the ecology and geographic distribution of *D. tejensis*, and provides an updated key to the Nearctic members of *Dielis*.

## 2. Materials and Methods

### 2.1. Collections

Field studies were conducted from 2023 through 2025 in the gently undulating terrain of the Brazos Valley in Texas, west of the Brazos River (Burleson Co., Washington Co., elevation 100–160 m and 75 m, respectively) and further south and southeast on the Gulf Costal Plains (Colorado Co., Harris Co., elevation 50 m and 10 m, respectively). This area is characterized by a diverse combination of open woodland, savanna and grassland ecosystems on sandy and loamy soils with clay-loam on the bottomlands. The prevailing climate is humid subtropical (*Cfa* in the Köppen classification) with long hot summers, mild winters and annual rainfall not exceeding 40 inches.

Scoliidae specimens were collected using an aerial net. In addition, museum specimens from the following collections were examined: Texas A&M University Insect Collection (TAMUIC, College Station, TX, USA), Louisiana State Arthropod Museum (LSAM, Baton Rouge, LA, USA), Florida State Collection of Arthropods (FSCA, Gainesville, FL, USA), and Smithsonian National Museum of Natural History (NMNH, Washington, DC, USA). The voucher specimens of *D. tejensis* were deposited in the above-mentioned institutions.

### 2.2. Geographic Distribution and Nomenclature

Data on the occurrence of the species in question came from current field surveys, the labels of museum specimens stored at TAMUIC, LSAM, and FSCA, and datasets provided by the Global Biodiversity Information Facility (GBIF, https://gbif.org/), and iNaturalist (https://inaturalist.org/) (research-grade records that were additionally verified based on the accompanying photographic evidence). They were plotted on OpenStreetMap using QGIS 3.40 (https://qgis.org/). Scoliidae nomenclature followed the one used by Osten (2005) [1] in his world catalog of this family. The plants were identified using the Flora of North America key (https://eFloras.org) and an updated key to the Texas taxa of *Hymenopappus* L′Hér. [26]. Plant nomenclature is consistent with that used in both keys and the World Flora Online database (https://worldfloraonline.org/, accessed in 2025).

### 2.3. Phenotypic Analysis

The external phenotypes of wasps were analyzed using Zeiss Stemi SV11 stereomicroscope (Zeiss, Oberkochen, Germany) equipped in Zeiss Axiocam 705 color camera. The morphological terminology follows the Anatomy ontology for Hymenoptera [27] as well as Betrem and Bradley [28]. The metasomal tergal band areas were measured from their images using ImageJ 1.46r software (https://imagej.net/ij/). Statistical analyses were performed using nonparametric Mann–Whitney in GraphPad Prism 10.6.0 (GraphPad Software, Boston, MA, USA).

### 2.4. Mitochondrial DNA Isolation and Sequencing

*D. tejensis* mitochondria were isolated from the thoracic muscles of a single wasp specimen using Qproteome Mitochondria Isolation Kit (Qiagen, Valencia, CA, USA). Mitochondrial DNA (mtDNA) was extracted from purified mitochondria by the phenol–chloroform method. To further reduce the possibility of accidental sequencing of mtDNA segments integrated into nuclear genome, trace amounts if any of nuclear DNA were removed from the mtDNA preparations by digestion with Exonuclease V (New England Biolabs, Ipswich, MA, USA). The mitochondrial genomes, with the exception of their regulatory region, were amplified in two pieces by long-range PCR using *Dielis*-specific primer pairs: *nad4*F 5′-TTTCTGATTACCTAAGGCTCATGTGTGA-3′/*cox2b*R 5′-TACCTCT-TACCTCCCGTTGAAGTATTGA-3′ and *rrnS*F 5′-TGAATCATTAAACCGTATTGACA-TGGCG-3′/*nad5*R 5′-ATGAACTAAAGCTGATACTGGTGTGGGA-3′, and LA Taq DNA polymerase (Takara Bio, Kyoto, Japan). The PCR conditions included incubation at 94 °C for 30 s followed by 62 °C for 11 min, which was repeated 30 times. The 11.9 and 7.1 kb large PCR products were purified on Amicon 30K filters (Merck Millipore, Cork, Ireland) and Nanopore-sequenced by Quintara Biosciences (San Francisco, CA, USA). The sequences of the repetitive regions were verified by Sanger sequencing.

## 3. Results

### 3.1. D. tejensis, Female Nova

**Figure 1:**
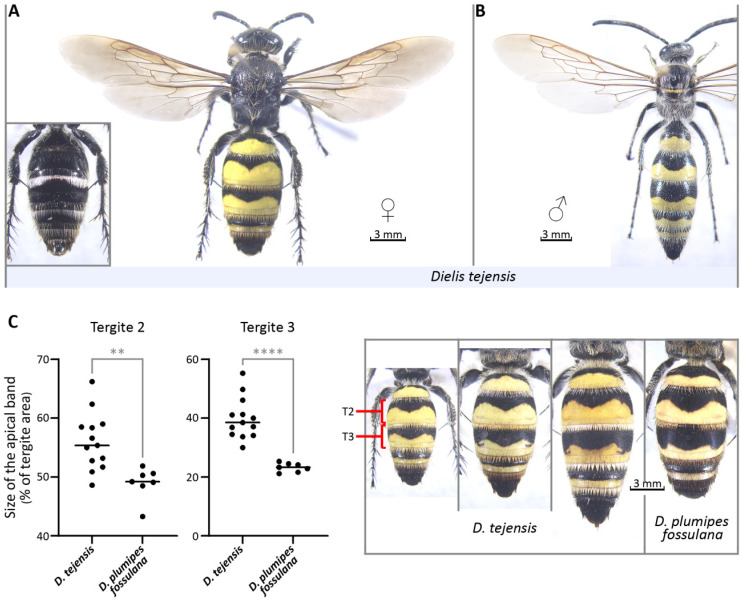
Habitus of *D. tejensis*. (**A**) Female alloreferent (inset: ventral view of metasoma); (**B**) male; (**C**) comparison of the sizes of the metasomal tergal apical yellow bands in female *D. tejensis* and *D. plumipes fossulana*. The areas of the tergal bands T1–T3 in *D. tejensis* are significantly larger than the corresponding areas in *D. plumipes fossulana* (Mann–Whitney test, ** *p* < 0.01, **** *p* < 0.0001, the horizontal lines represent median values).

Figure 1; urn:lsid:zoobank:org:pub:7B56A179-38E5-4EA7-9CFE-388B0C62EE38.

Female *D. tejensis* were collected from the inflorescences of plants frequently visited also by males of this species. West of the Brazos River, *D. tejensis* was the only Scoliidae species observed, apart from occasional presence of *Colpa octomaculata* (Say). A little further to the southeast, in Harris Co., *D. tejensis* was not so rare just a few years ago, co-existing alongside the much more numerous *D. plumipes fossulana* and *Scolia bicincta* Fabr. Most recently, only one female of *D. tejensis*, as well as a dozen or so specimens of *Scolia dubia* Say, have been found there.

*D. tejensis* females were identified by comparing their mtDNA sequences with those of males (NCBI GenBank MN990424 and of a few newly collected specimens). In pairwise comparisons, the intraspecific differences between mtDNA coding regions of the females were insignificant, i.e., <0.2%, whereas the interspecific difference between *D. tejensis* and the closely related *D. plumipes fossulana* (GenBank KT740996) was ~7%, as previously reported for the males [15]. Similarly to *D. tejensis* males, female mtDNA had an enlarged *nad2-trnW* intergenic region compared to *D. plumipes fossulana*, a shortened insert dividing the *cox2* gene into two complementary genes, *cox2a* and *cox2b*, and a TAA stop codon in the middle of this insert, in-frame with *cox2a* and *cox2b*.

*Materials examined*: *D. tejensis*: USA ● Texas, Burleson Co.; between 30.5516° N, -96.7989° W, elevation 153 m and 30.5537° N, -96.7310° W, elevation 100 m; 21 Mar. 2023–27 Sept. 2025; 12 ♀♀ (2 ♀♀ of the 2nd generation were released back to their natural environment) and 54 ♂♂; author leg.; ● Texas, Colorado Co.; 29.6720° N, -96.2694° W, elevation 50 m; 19 and 27 Sept. 2025; 10 ♂♂; author leg.; ● Texas, Harris Co., Houston; 29.76° N, -95.44° W, elevation 14 m; 23 Sept. 2023; 1 ♀; author leg.; 05 Sept. 2013 and 15 Oct. 2014; 3 ♂♂; author leg.; ● Texas; 14 ♂♂ in TAMUIC (more details are provided in Table S1); ● *D. plumipes fossulana*: USA ● Texas, Harris Co., Houston; 29.8° N, -95.4° W; 21 Sept. 2013; 8 ♀♀ and 2 ♂♂; author leg.; ● Texas and Florida; 14 ♂♂ in TAMUIC (more details are provided in Table S1) ● Louisiana; 3 ♂♂ in LSAM (more details are provided in Table S1).

*Description*: **Body** length: 17–27 mm (median: 19 mm, n = 13). **Forewing** length: 13–20 mm (median: 15 mm, n = 13). **Head**: clypeus slightly projected medially, its discal region slightly convex, impunctate but irregularly vertically striated, not separated from lateral lobes by anterior notches, these lobes in turn covered with dense coarse punctuation with long erected setae that contrast with short appressed pubescence along the anterior edge of the clypeus except for its central part; scrobes mostly smooth with appressed pubescence; laminae frontalis slightly elevated; spatium frontale densely punctate except for narrow impunctate irregular median line; frons punctuation extends laterally to eye lower lobes and sinuses and above spatium frontale where it becomes gradually more sparse irregular and finally almost disappears around the ocelli; ocular sinuses with a weak internal depression; frontal ocellus in a shallow depression; vertex largely smooth with few scattered punctures and few setae, becoming dense and fine punctate only on its declivous portion along the carina occipitalis; tempora similarly punctate with erect setae; gena largely smooth with few scattered punctures of different size and mostly without setae, but becomes densely and finely punctate along the hypostomal carina; occiput densely and fine punctate except for its smooth half adjacent laterally to occipital carina, with dense setae; postocciput densely and finely punctate along lateral and ventral part of the foramen. Antennae: scape and pedicel punctate, scape more densely on its basal half where it is covered with erect setae, pedicel covered with smaller punctures than those on scape, last flagellomer is the longest of all flagellomers. Mandibles: outermost furrow not visible in lateral view. **Mesosoma**: pronotum densely punctate and covered with dense erect setae except for its margins, the anterior vertical portion with the neck-like projection and the lateral lower areas; mesoscutum coarsely and irregularly punctate, less densely in its central and posterior areas, with parapsidal furrows arising from its posterior margin and lateral and posterior areas of the disk slightly dented; scutellum sparsely and irregularly punctate with setae arising from punctures; metanotum irregularly punctate, mostly on the anterior half of its central sector, with setae arising from punctures; propodeum tripartite by longitudinal ridges, its dorsal surface densely punctate except median glabrous stripe and anterior parts of areas horizontales laterales next to spiracles, the transition of the dorsal surface of propodeum to its posterior vertical and slightly concave portion angulate with a ridge, area horizontalis medialis weakly produced medially, area posterior medialis impunctate except along its upper margin; areae posterioles laterales impunctate on the sides next to area posterior medialis and on spiracular areas; areae laterales smooth along metapleuron; a carina posterior to the spiracle, between areae horizontales laterales and areae laterales, present; propodeal setae long erected and follow punctuation pattern, area posterior medialis covered with short appressed pubescence; mesopleuron elevated longitudinally with rounded vertical ridge crossed by transverse furrow and with high elevation at its upper end forming platform, punctate on its anterior half along the ridge, on lower plate of the posterior half and on its elevated upper platform, punctures with long erect setae except for short pubescence on the upper platform; metapleuron divided by a horizontal suture into largely impunctate upper and lower plates, upper plate with horizontal narrow sector with fine and dense punctures and short appressed pubescence, delimited by a sharp crest from the metapleural disk; mesosternal and metasternal plates almost flat with a weak longitudinal central keel. Wings: forewing with two submarginal cells and two recurrent veins, hyaline, with their surface mostly bare except for the area along their anterior margin, which includes stigmatal and parts of the subcostal, first submarginal and marginal cells. Legs: tibial spur formula 1-1-2, hind tibial spurs spatulate, spines on the outer face of hind tibiae emerging from transverse ridges. **Metasoma**: tergites T1–T3 dull, T1 without a median tubercle, irregularly punctate, denser on the anterior declivous side, especially on its narrowed base, with horizontal irregular median and apical rows of punctures, and long erect setae except for the discal area, T2–T5 with subapical and apical rows of punctures with long setae forming fringes, significantly longer subapically but more dense apically, otherwise T2–T4 with short and sparse setae that become longer and denser laterally, T5 punctate with relatively long setae in addition to the fringes, truncated surface of T6 reticulate longitudinally and with short mostly appressed thick and dense bristles; ventral side of metasoma shiny and flat along midline, metasomal sternite S1 mostly impunctate with sparse and fine punctuation and some erect setae along its sides, S2 without a median tubercle, S2–S5 irregularly punctate with very fine dense punctuation on the anterior half and larger punctuation posteriorly and laterally, with subapical and apical rows of punctures with bristles forming fringes, much more dense apically, hypopygium irregularly punctate with dense short bristles along its sides and apically and two lateral spine-like projections largely concealed by bristles. **Pigmentation**—Head black; mandibles black with longitudinal brown stripes; mesosoma black; legs mostly black with dark brown tarsi; wing membrane brownish with some weak violet reflection, slightly more infuscate beyond the cells and within first submarginal and marginal cells, veins brown; metasomal apical T1–T4 bands yellow and complete, T1–T3 bands covering 39–55% (medians, n = 13) of the tergite surface, T4 band limited to the area between subapical and apical rows of setose punctures, T2 and T3 bands with central, sharply pointing notch and usually rudimentary side notches; vestiture: erected setae on front of the head and mesosoma pale yellow to light orange (pronotal collar), pale yellow or white on clypeus, white on gena, ventral and lateral surfaces of mesosoma and metasoma, except S5 and S6 where they are pale brown, white on T1 of metasoma; appressed pubescence of clypeus and on propodeal area posterior medialis silvery; apical fringes on T1 white, on T2–T4 yellow to pale orange, on T5 and T6 brown, fringes on S2-S4 white to pale yellow, those on S5 pale brown; wing setae brown; setae on legs white; spurs dark brown. **Variation**—The variability of the females is relatively small and mainly concerns body size, tergal band size and the shape of tergal band notches (Figure 1). Some specimens have a widened central notch on the T2 band. Rarely, lateral notches on T3 are developed more like those on *D. plumipes fossulana*. Moreover, the color of the dorsal vestiture on the head and thorax, and T2–T4 fringes can vary from pale yellow to pale orange or pale brown, and sometimes the forelegs are dark brown.

*Diagnosis*: The female *D. tejensis* (Figure 1A) differs from the male (Figure 1B) in having a more robust body structure, short antennae with 10 flagellomeres, head, mesosoma and legs all black without yellow markings, forelegs strongly adapted to digging in the soil, hind tibial spurs spatulate and dark brown, more tinted wings, metasoma with six visible segments, of which the first one is as wide as the propodeum, metasomal tergites T1–T3 matt, only T1–T4 with apical yellow bands, metasomal sternites without yellow bands or side stripes, hypopygium with two short lateral spines, and dorsal vestiture mostly pale yellow to pale orange (not white like in males). From the females of other Nearctic *Dielis* species, the female *D. tejensis* differs in having slightly more densely punctate disks of the mesoscutum and scutellum, a feature that is, however, quite variable and becomes more apparent when comparing longer series of specimens. Compared to closely related and partially sympatric *D. plumipes fossulana* (Figure 1C), the vestiture on the dorsal side of the head and mesosoma is paler and less orange, resembling in this respect the northern subspecies of *D. plumipes*: nominate and *confluenta* (Say); fringes on metasomal tergites T2–T4 are also more pale yellow and less orange or brown (the difference is especially visible on T4); fringes on sternites S2–S4 are white; T1–T3 yellow bands are wider, more like in the nominate and *confluenta* subspecies of *D. plumipes* (T2 by 11%, *p* < 0.01; T3 by 39%, *p* < 0.0001). The central notch, especially in the T2 band, is usually more acute, and the lateral notches on the T3 band are usually more-or-less rudimentary, again resembling more, in this respect, subspecies of *D. plumipes* from the northern part of its range. However, the female *D. plumipes confluenta* differs from *D. tejensis* in having the wrinkled surface of the area posterior medialis of the propodeum and often an incomplete yellow band on T4 or lacking it altogether, while the female of the nominate subspecies of *D. plumipes* differes from *D. tejensis* in that the posterior margin of the dorsal part of its propodeum does not form an overhanging ledge. It is important to remember, however, that some of the diagnostic features presented may become blurred in melanistic forms or in some specimens originating from areas at the intersection of the ranges of the *D. plumipes* subspecies. In cases where the species affiliation based on phenotypic characters seems uncertain, Sanger sequencing of mtDNA should be considered. Examples of primers that can be used to amplify short but still informative fragment of *Dielis* mtDNA for sequencing are 5′-TCGTATAGAACTAGGAATAGCAGGATC-3′ and 5′-TCCAGTTCCAATTCCTTTTCCTACA-3′.

### 3.2. Seasonal Phenotypic Variability and Bionomics of D. tejensis

The seasonality of *D. tejensis* was monitored at Brazos Valley locations (Burleson Co.) crossing the study area over a distance of 10 km. *D. tejensis* appeared to have likely more than one generation per year, being more common in spring, especially in April, and in the second half of summer and autumn, from July through October (Figure S1). A similar fluctuation in the frequency of *D. tejensis* observations across its entire range is shown in iNaturalist (https://inaturalist.org/taxa/1475187-Dielis-tejensis/, accessed on 27 April 2025). It is characterized by the presence of three main peaks of occurrence in April, July–August, and October.

**Figure 2:**
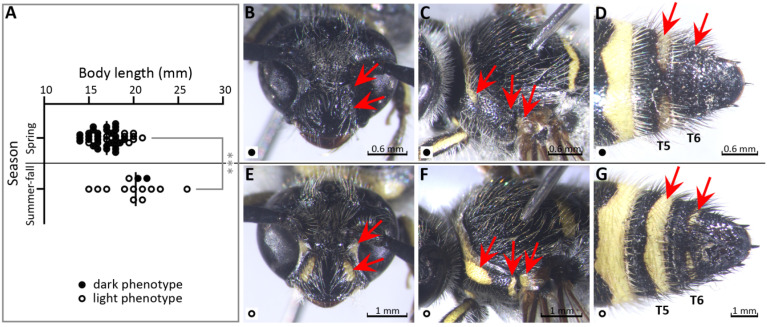
Apparent seasonal polyphenism in male *D. tejensis* (Brazos Valley). Red arrows point to the presence or absence of selected phenotypic features. (**A**) Differences in body size and integument pattern (clypeus entirely black ● versus black with yellow side stripes or spots ○) between the spring and summer–autumn cohorts (Mann–Whitney test, *** *p* < 0.001, the horizontal line represents the median value). (**B**–**D**) Darker spring phenotype. (**B**) Head in front view with an entirely black clypeus and scrobes. (**C**) Mesosoma with a small humeral yellow spot and without a yellow spot at the posterior edge of the pronotal lobe; the basal yellow spot on the tegula is barely recognizable. (**D**) Apical part of metasoma with a yellow band on tergite T5 weak and interrupted in the middle. (**E**–**G**) Lighter summer–autumn phenotype. (**E**) Head in front view with a clypeus featuring a yellow stripe on each side (often reduced to dots in spring forms) and yellow scrobes. (**F**) Mesosoma with a large and elongated humeral yellow spot and with a yellow elongated spot along the posterior edge of the pronotal lobe; the basal yellow spot on the tegula is clearly visible. (**G**) Apical part of a metasoma with a well-developed yellow band on T5 and a residual interrupted apical yellow band on T6.

Of interest, the summer–autumn generation(s) of this species featured almost complete absence of adult female wasps at any of the monitored locations. This phenomenon correlated with the existence of seasonal variability of *D. tejensis* phenotype in males. It manifested itself in the existence of differences in body size and the degree of development of the yellow pattern between the spring and summer–autumn cohorts (*p* < 0.001 for both comparisons, n = 40 and 14, respectively) (Figure 2). In spring, the wasps were on average 16% shorter than in summer–autumn, and had a reduced yellow pattern, including the entirely black clypeus in 73% of specimens (a feature used in the statistical evaluation of the difference in color pattern) (Figure 2A,B), a reduced yellow pigmentation of the strobes (Figure 2B), weaker yellow stripe along the posteriol edge of the pronotum continuous with weaker humeral spots, a lack of yellow stripes along the posterior edges of the pronotal lateral lobes (Figure 2C), a reduced or missing basal yellow spot on the tegulae (Figure 2C), a narrowed and sometimes interrupted apical yellow band on T5 (Figure 2D) and occasional loss of the S5 band (18%). In summer and autumn, smaller or melanotic forms constituted only ~14% of the Brazos Valley population. Most males at this time had yellow stripes or spots on the sides of their clypeus (Figure 2E), elongated humeral spots on the pronotum (Figure 2F), and basal yellow spots on the tegulae (Figure 2F). In addition, 25% of the summer–autumn generation(s) had a residual narrow apical yellow band on T6 (Figure 2G) and 15% had a more-or-less developed yellow stripe on the outer surface of the middle tibiae. As could be expected, lighter-colored TAMUIC and FSCA museum specimens of *D. tejensis*, dating from the 1930s to the present day, comprised the majority of specimens collected in the summer–autumn period. Sequencing of the mtDNA did not reveal any significant differences between the darker and lighter forms (pairwise difference for coding regions of mtDNA < 0.2%). However, the described seasonal phenotypic changes do not seem to be equally strongly expressed throughout the species’ range. At sites located further south (Gulf Coastal Plains in Colorado Co.), no more than half of male wasps of the summer–autumn generation(s) had the yellow stripes on the clypeus, their humeral spots on the pronotum were less elongated compared to those of the Brazos Valley wasps, and the yellow stripe on the outer side of the medial tibiae was always absent (pairwise differences between mtDNA of those and Brazos Valley specimens ≤ 0.1%, n = 10; data regarding the spring cohort were not collected at this location).

Female *D. tejensis* were observed mainly in spring, most frequently in the second half of April. In summer and autumn, they were observed only individually and sporadically, and their seasonal variability requires further investigation.

**Figure 3:**
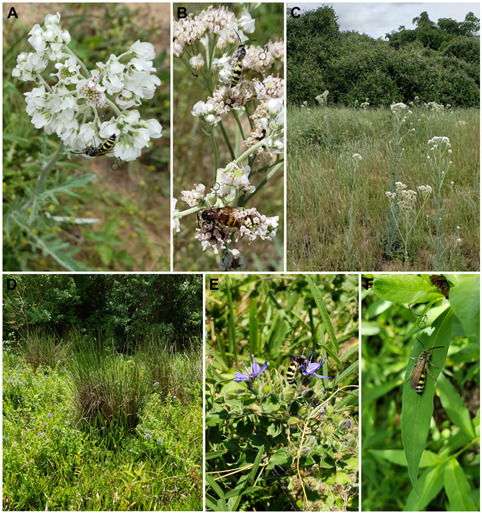
Selected floral associations and habitats of *D. tejensis*. (**A**,**B**) *D. tejensis* males and a female on the inflorescences of *H. artemisiifolius*; (**C**) April meadow on sandy soil, with a significant share of flowering *H. artemisiifolius*, often visited by *D. tejensis*; (**D**) a wetland plant community with blooms of *Hydrolea* and *Persicaria* frequently visited by *D. tejensis* at the turn of summer and autumn; (**E**) *D. tejensis* male on flowers of hydrophilic *H. ovata* in September; (**F**) a male *D. tejensis* resting in the shade on a leaf of *P. hydropiper* in August.

The summer–autumn resurgence in the population of *D. tejensis* was also associated with changes in the environment, including plant communities visited by imagines. In March, *D. tejensis* frequented *Rubus trivialis* Michx. (Rosaceae) and *Vicia ludoviciana* Nutt. (Fabaceae) in dry locations. Later, in April, at the height of the *Hymenopapus artemisiifolius* DC. (Asteraceae) flowering season, the wasps were observed almost exclusively on the inflorescences of this biennial herb (Figure 3A,B), growing among loose vegetation on well-drained sandy soil at the edges of pine–oak open woodlands, in savannas, and on prairies (Figure 3C). The end of the flowering period of *H. artemisiifolius* in May was correlated with a significant decrease in the number of *D. tejensis*. During this time, this wasp was observed only occasionally, mainly on *Monarda* spp. (Lamiaceae). However, from July to October, when the air temperature in the shade was constantly above 35 °C, *D. tejensis* was observed again in greater numbers, but on adjacent, local depressions with more water-saturated soil (Figure 3D). The imagines (males) were feeding on the flowers of hydrophiles such as *Hydrolea ovata* Nutt. ex Choisy (Hydroleaceae) (an obligate wetland plant (OBL)) (Figure 3E), *Persicaria hydropiper* (L.) Delabre (Polygonaceae) (OBL), and later on *Pluchea odorata* (L.) Cass. (Asteraceae) (a facultative wetland plant (FACW)) and *Eupatorium serotinum* Michx. (Asteraceae) (more versatile FAC plant). In addition, they were observed resting in loose groups, in the shade of nearby bushes and trees, on the leaves of *P. hydropiper* (Figure 3F) and other plants. *D. tejensis* showed similar habitat preferences during late summer and autumn for areas closer to the Gulf coast (Colorado Co., Harris Co.), where it was found near shady places along creeks and other water bodies on *E. serotinum*, *P. hydropiper*, and *Micania scandens* B.L.Rob (Asteraceae) (FACW).

### 3.3. Geographic Distribution of D. tejensis

**Figure 4:**
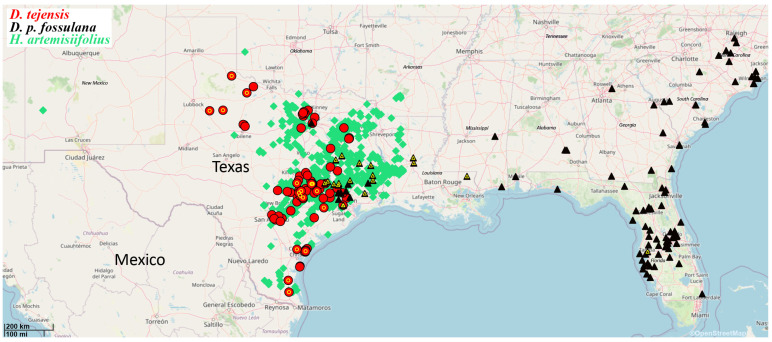
Overlap in the geographic ranges of *D. tejensis* (red dots) and one of the main food-plants for its imagines, *H. artemisiifolius* (green diamonds). The black triangles indicate the locations where *D. plumipes fossulana* occurred. The localities of *D. tejensis* and *D. plumipes fossulana* from which the original specimens were seen by the author are marked with a yellow circle or triangle, respectively. Sources of the presented data: current field studies, museum specimens, GBIF and iNaturalist databases (see Section 2 and Section 3 for details); base map image: ©2025 OpenStreetMaps and its data providers.

New information regarding the occurrence of *D. tejensis* came from current field studies, museum specimens preserved at TAMUIC, LSAM, and FSCA, and from the iNaturalist database (https://inaturalist.org/taxa/1475187-Dielis-tejensis/, accessed on 6 December 2025 and verified from images of the males). The range of *D. tejensis* that emerged from all the available data covers approximately the eastern half of Texas, especially a broad belt of Post Oak Savannah and Blackland Prairies (EPA Ecoregion 6), running south from Oklahoma to San Antonio and the Gulf coast (Figure 4; Tables S1 and S2). The eastern half of *D. tejensis* range and western edge of *D. plumipes fossulana* range (based on current field studies, TAMUIC, LSAM and FSCA museum specimens, GBIF.org (24 December 2025) GBIF Occurrence Download https://doi.org/10.15468/dl.5rtge3 and iNaturalist (https://inaturalist.org/taxa/1063323-Dielis-plumipes-fossulana/, accessed on 27 December 2025 and verified from images of the males)) overlap in areas where savannas transition into the Piney Woods in East Texas (Figure 4; Tables S1 and S2). However, while *D. plumipes fossulana* was a relatively common and dominant species in, e.g., the Houston area (most recently in the first decade of this century), it was rarer farther northwest where *D. tejensis* is currently a dominant *Dielis* species.

Notably, the range of *D. tejensis* coincided closely with the range of one of its main spring nectaring plants, *H. artemisiifolius* (current field studies, and GBIF.org (22 December 2025) GBIF Occurrence Download https://doi.org/10.15468/dl.n4pv5x) (Figure 4). Most of the *D. tejensis* locations were mapped in the western two-thirds of the *H. artemisiifolius* range. The eastern one-third of the *H. artemisiifolius* range lies in the area covered by denser forest complexes, which *D. tejensis* avoids. *H. artemisiifolius* was the only *Hymenopappus* species found at *D. tejensis* localities.

It seems that the similarity in distribution ranges of *D. tejensis and H. artemisiifolius* may be due to both the nutritional attractiveness of *H. artemisiifolius* flowers for *D. tejensis*, including its females which are present in the largest numbers during the flowering period of *H. artemisiifolius*, and the similarity of other environmental requirements of both organisms. The range of *D. tejensis* may also depend on the range of the beetle host of its larvae. Although this host remains unknown, one of the Scarabaeidae observed together with *D. tejensis* on *H. artemisiifolius*, namely *Trichiotinus texanus* (Horn) (Cetoniinae), has a range (https://bugguide.net/node/view/55942/image/, accessed on 1 March 2026) quite similar to that of *D. tejensis*. The larvae of this species are, however, somewhat smaller even than those of Rutelinae, which are among the smallest hosts of Scoliidae of a similar size to *D. tejensis*. For this reason, a more likely candidate for the host of *D. tejensis* might be *Strigoderma arbicola* (Fabr.) (Rutelinae), whose aggregations on Rosaceae flowers have been observed, especially in spring, near *D. tejensis* locations. However, this species’ range covers the entire eastern half of the USA and cannot explain the distribution of *D. tejensis*.

### 3.4. Key to Nearctic Species of Dielis

So far, several regional keys for identifying Scoliidae occurring in North America have been published [23,29,30,31,32,33], but only the oldest of them (Bradley 1928, [29]) included all the *Dielis* species (under the genus name *Campsomeris*) known at that time from the Nearctic realm. The key shown below is a modification of Bradley’s key [29] to include *D. tejensis*, the only addition to *Dielis* from the Nearctic in the last 100 years.

1 Antennae short, with 10 flagellomeres; metasoma with six visible segments, its basal segment as wide as the propodeum, hypopygium with two short lateral spines (females)......................................................................................................2

– Antennae long, with 11 flagellomeres; metasoma composed of seven visible segments, its basal segment narrower than propodeum, hypopygium with three long, apical spines projecting beyond the end of metasoma (males)...............................9

2(1) Pronotum usually with humeral round yellow spots; middorsal part of propodeum not longer than metanotum, rounding off into entirely punctate vertical area posterior medialis (mainly southwestern United States and northern Mexico, including Sonoran ecozone)....................................................................................*pilipes* (Saussure)

– Pronotum without yellow spots; the transition between the middorsal part of the propodeum and vertical area posterior medialis is abrupt, often in form of a ridge, area posterior medialis impunctate, except along its upper margin (*plumipes* species-group *sensu* Bradley, 1964)............................................................................3

3(2) Metasomal bands orange to red (sometimes yellow or orange band on T1 in *D. tolteca*), bands on metasomal tergites T2 and T3 occupying almost the entire tergite surface.........................................................................................................................................4

– Metasomal bands yellow and narrower, rarely occupying more than 2/3 of the tergite surface......................................................................................................5

4(3) Tergites T2, T3 almost entirely orange to red, sometimes orange-red band on T4 (usually between apical and subapical fringes); wings dark brown (Mexico, Central and most of South America, and the Caribbean region, introduced in Florida and Louisiana, established in Florida).....................................................*dorsata* (Fabricius)

– Tergites T2, T3 almost completely and T4, usually between apical and subapical fringes, orange to dark orange, sometimes also yellow or orange apical band on T1 (form *dives* Provancher); wings darkened but somewhat less than those in *D. dorsata* (Lower Sonoran ecozone from the eastern USA to South-Central Texas, Mexico, El Salvador, Honduras, Nicaragua, and Haiti).................................*tolteca* (Saussure)

5(3) Humeral bristles white or nearly so; metanotum smooth and polished, at most sparsely punctate, devoid of bristles, sometimes with a transverse yellow bar; metasomal band on T4 broken or absent; metasomal sternites S2 and often S3 with apical yellow bands reduced to lateral stripes (Florida and the Caribbean region; ssp. *nassauensis* Bradley in the Bahamas)...........................................*trifasciata* (Fabricius)

– Humeral bristles pale yellow to dark orange; metanotum punctate hirsute and entirely black; metasomal band on T4 present or absent; metasomal sternites entirely black.........................................................................................................6

6(5) Pronotal vestiture dark orange; metasomal dorsal bristles, except those on the last two apical segments, pale orange; apical and subapical fringes on T3–T4 brown; yellow bands on T1–T4 relatively narrow, band on T3 occupies up to 1/4 of the tergite surface and features wide and shallow median notch and slightly deeper lateral notches on each side (Austroriparian ecozone from North Carolina to eastern Texas)...................................................................*plumipes fossulana* (Fabricius)

– Pronotal vestiture pale yellow to pale orange; metasomal dorsal bristles, except those on the last two apical segments, white and yellow to pale orange; apical and subapical fringes on T3–T4 pale yellow, pale orange or brown; yellow bands on T1–T3 wider, T2 band occupying from half to 2/3 of the tergite surface and featuring usually acute median notch, T3 band occupying up to almost half of the tergite surface and featuring usually insignificant or no lateral notches....................................................7

7(6) Area posterior medialis of propodeum usually very coarsely rugose, T4 band often incomplete or absent (plains of Upper Austral ecozone between Appalachian and Rocky Mts)........................................................................*plumipes confluenta* (Say)

– Area posterior medialis of propodeum smooth, T4 band always present and complete........................................................................................................8

8(7) Posterior margin of the dorsal surface of propodeum not forming an overhanging ledge, having instead “a median projecting point carried laterad by a short carina” [29] (Upper Austral ecozone from Massachusetts to Georgia and Kentucky, east of the Appalachian Mts)..................................................................*plumipes plumipes* (Drury)

– Dorsal surface of propodeum forming an overhanging ledge or shelf, similar to that of, e.g., *D. plumipes fossulana* or *D. plumipes confluenta* (from Oklahoma through East Texas to the Gulf Coast)..........................................................*tejensis* Szafranski

9(1) Frons divided by the longitudinal groove nearly reaching the spatium frontale, flanked by glabrous, impunctate tubercles devoid of bristles; eye margins entirely black; mandibles entirely black–brown; pronotum usually without transverse yellow stripe along the posterodorsal margin but with round yellow spots within each humeral angle; pro- and mesotibiae with a longitudinal yellow stripe on the dorsal side; propodeum rounding from behind the metanotum into the posterior area; all vestiture on the metasomal segment V white (note that the presence of a yellow band on the segment V is no longer specific to this species) (mainly southwestern United States and northern Mexico, including Sonoran ecozone)...................*pilipes* (Saussure)

– Frons mostly with a weak median groove terminating in a pit, punctate and with erect bristles; usually yellow stripe along the inner edge of the eye lower lobe, often spreading on the scrobes; mandibles yellow at the base; pronotum without or with yellow stripe along its posterior margin; at least protibiae with yellow stipe dorsally; dorsal area of the propodeum well defined, its midsection longer than that of metanotum; vestiture of the metasomal segment V black except for the white setae originating from apical yellow band in *D. tejensis* (*plumipes* species-group *sensu* Bradley, 1964).................................................................................................10

10(9) Eye outer margin often with narrow yellow stripe; pronotum with a yellow stripe along its posterior edge continuous with humeral spots that usually extend uninterruptedly to the spots at the edges of the pronotal lobes near tegulae; at least pro- and mesotibiae with yellow stripes on the dorsal side; femora usually with a yellow stripe......................................................................................................11

– Eye outer margin usually entirely black; pronotum entirely black or with a posterior median yellow stripe continuous with humeral spots that, however, even if elongated, do not reach spots on the pronotal lobes next to the tegulae; usually only protibiae with a yellow stripe on their dorsal side; femora usually black, at most with apical yellow spot......................................................................................13

11(10) Clypeus either entirely yellow or yellow with black central spot; metasomal sternite S2 band broad, S2 and usually S3 bands uninterrupted medially (Florida and the Caribbean region; ssp. *nassauensis* Bradley in the Bahamas)........*trifasciata* (Fabricius)

– Clypeus black with yellow lateral margins; sternite apical bands S2–S4 narrow and interrupted medially.................................................................................12

12(11) Forewing setae restricted mostly to subcostal, stigmatal, marginal and submarginal cells; harpe with a thin row of setae (Mexico, Central and most of South America, and the Caribbean region, introduced in Florida and Louisiana, established in Florida)....................................................................................*dorsata* (Fabricius)

– Forewing membrane usually more broadly setose, also on its anterior half beyond cells; harpe with a brush of relatively long prominent setae (Lower Sonoran ecozone of the USA, Mexico, El Salvador, Honduras, Nicaragua, and Haiti).*tolteca* (Saussure)

13(10) Pronotum with well-defined humeral spots that, however, do not reach the posterior edges of pronotal lobes near the tegulae, but are linked together by a yellow stripe along posterodorsal edge of pronotum; metasomal segment V with apical yellow band broadly interrupted medially on the sternite; in addition, a residual widely interrupted apical T6 band is sometimes present (from Oklahoma through East Texas to the Gulf Coast)..................................................*tejensis* Szafranski

– Humeral spots on pronotum absent or inconspicuous, dorsal face of pronotum at most with a weak median yellow stripe along its posteriol edge; metasomal segment V usually entirely black................................................................................................14

14(13) Pronotum sometimes with a weak posterior yellow stripe; yellow bands on T2 and T3 relatively narrow, T2–T4 bands with broad and shallow median and lateral emarginations, S2–S4 bands narrowly interrupted in the middle, tergites with a brilliant deep blue iridescence (Austroriparian ecozone from North Carolina to eastern Texas).................................................................................*plumipes fossulana* (Fabricius)

– Pronotum typically without posterior yellow stripe; yellow bands on T1–T3 wider than those on *D. plumipes fossulana*, T2–T4 bands with narrower median notches and rudimentary or relatively less developed lateral notches, S2–S4 bands widely interrupted medially, tergites with a more greenish reflection..........................................15

15(14) Occurs in the Upper Austral ecozone from Massachusetts to Georgia and Kentucky, east of the Appalachian Mts...........................................*.plumipes plumipes* (Drury)

– Occurs on the plains of the Upper Austral ecozone between Appalachians and Rocky Mts..................................................................*plumipes confluenta* (Say)

## 4. Discussion

The limited distribution area of *D. tejensis* and the significant mutual phenotypic similarity of the *Dielis* species undoubtedly contributed to the late recognition of *D. tejensis* as a separate species (in the past, it was most often treated as *D. plumipes fossulana*). Likely for the same reasons, the female *D. tejensis* remained unknown until now. Identifying the female *D. tejensis* was further complicated by its greater rarity compared to males and the strongly pronounced sexual dimorphism in *Dielis* and other Campsomerini.

The female *D. tejensis* shows all the features typical of *Dielis sensu stricto*, such as an entirely black head, pronotum, and mesonotum, a considerably punctate mesopleura with a visible vertical ridge, unpunctuated area posterior medialis of the propodeum, matt metasomal tergites with yellow apical bands, and spines on the outer face of the hind tibia that emerge from transverse ridges. At first glance, it can be distinguished from partially sympatric and closely related *D. plumipes fossulana* by its lighter vestiture, including pronotal bristles and the metasomal fringes on tergites T2–T4, and wider apical yellow bands on T1–T3. From other subspecies of *D. plumipes*, living further north and northeast, female *D. tejensis* differs especially in the sculpture and shape of the propodeum. However, these phenotypic differences can sometimes become blurred, and in such cases, Sanger sequencing of a fragment of mtDNA may prove helpful.

Based on the frequency of *D. tejensis* sightings throughout the year, it seems that this species is at least bivoltine. Among the Nearctic *Dielis*, *D. tolteca* also exhibits a somewhat similar trait (https://inaturalist.org/taxa/893824-Dielis-tolteca/, accessed on 27 April 2025). On the other hand, *D. plumipes* subspecies seem to primarily follow a univoltine mode of development, with the main flight period occurring in the summer (both northern subspecies) or summer and early autumn (*D. plumipes fossulana*) (https://inaturalist.org/taxa/893824-Dielis-tolteca/, accessed on 27 April 2025).

**Figure 5:**
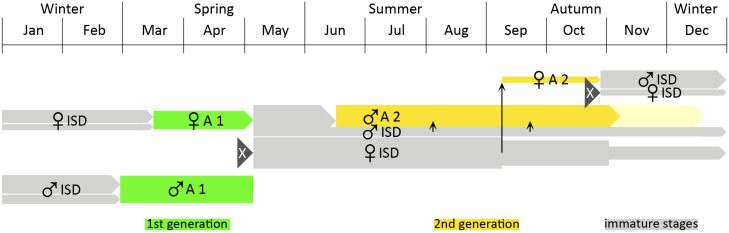
Diagram of the proposed developmental cycle of *D. tejensis*. Abbreviations: ISD—immature stage dormancy, A—adult wasp; the vertical arrows indicate the termination of dormancy by some larvae and the emergence of adult wasps. Females of the spring generation emerge later than males and are also less numerous than them (Figure S1). Unfertilized eggs from these females develop mostly into males of the second generation, while fertilized eggs develop into females. But before the latter happens, female immature stages enter a period of aestivation. Only some of them break this dormancy in the autumn and appear as a second generation of females (Figure S1). The offspring of these females overwinter as immature stages. According to this model, the wasps of the spring generation are therefore the mixed offspring of the first- and second-generation wasps from the previous year.

*D. tejensis* reemerges in the summer as an extremely male-biased second generation, perhaps due to the female immature stages entering aestivation or summer diapause. Female hymenopterans are generally larger than males, their larvae have higher nutritional requirements and are more sensitive to insufficient quantity or quality of food, thus often reacting by entering dormancy [34,35]. Likewise, high summer temperatures and drought can also trigger state of dormancy [35]. Clausen (1932) [8] mentions that in the tropics, the development of Scoliidae may be temporarily inhibited due to high temperatures or drought, resulting in aestivation. The region of North America where *D. tejensis* is found experiences both temperatures that frequently exceed 35 °C in the summer and early autumn and high humidity with low rainfall. However, although summer dormancy is beneficial for surviving unfavorable conditions, if it mainly concerns only one gender, in the long run, such a strategy may reduce the species’ chances of survival in a given area. A model of *D. tejensis* life cycle that explains its seasonality and differences in sex ratio between generations is proposed in Figure 5. According to this hypothesis, most females are univoltine due to entering the summer–autumn period of aestivation, which then transitions into hibernation lasting until the following spring. However, in autumn, a relatively small portion of female immature stages apparently interrupt their dormancy, complete development, and give rise to a new brood of males from unfertilized eggs and females mostly from fertilized eggs (e.g., [8]) which overwinter probably as pupae.

The reemergence of *D. tejensis* in the summer coincides with the phenotypic change visible in a significant fraction of male wasps. Experiments on *Drosophila* suggest that darker forms of *D. tejensis* may be the result of the exposure of the wasp prepupae or pupae to low temperatures in winter and that they may be better adapted to spring conditions compared to lighter forms (e.g., [36,37]). Because of the rarity of female *D. tejensis* in the second half of the year, it is currently unknown if or how this phenotypic variability manifests in them. Assuming that there are similar trends in phenotypic changes in both sexes, one can expect a fraction of second-generation summer–autumn females having, e.g., more strongly developed metasomal yellow bands. Seasonal phenotypic changes, occurring in response to changes in temperature, humidity or photoperiod, are known in several insect groups (e.g., [38]). In Hymenoptera, seasonal polyphenism was described in Symphyta [39], Cynipidae [40], Eulophidae [41,42], Vespidae [43], Formicidae [44,45], and Apidae [46,47].

The life cycle, featuring more than one generation per year or protracted emergence, entailed changes in the habitat and food plants visited by *D. tejensis* in spring versus the second half of the year (from drier to wetter environments, and from plants with facultative upland indicator to obligate and facultative hydrophiles), which might have facilitated *D. tejensis* differentiation from a univoltine ancestor. Perhaps the most intriguing trophic relationship involving imagines of *D. tejensis* is their association in spring with *H. artemisiifolius*. Both *D. tejensis* and *H. artemisiifolius* have closely overlapping geographic ranges that are restricted to the eastern half of Texas. Unless this is a purely coincidental overlap in ranges, it might indicate a relationship involving a wasp with specialized dietary preferences, except that *D. tejensis* is rather a polylectic species. Certainly, such similarity in ranges could also reflect a relationship between a scarabaeid host of the *D. tejensis* larvae and *H. artemisiifolius* or another plant with a similar range. Although the identity of this host remains unknown, one of the scarab beetles observed in spring on *H. artemisiifolius*, *Trichiotinus texanus*, has a range quite similar to that of *D. tejensis*. The range overlap may also indicate an antiquity of the *D. tejensis*–*H. artemisiifolius* association and co-evolution of both these organisms involving adaptation to prairie, savanna, and open-forest environments in climate characterized by high average annual temperatures, humidity, and relatively small yearly precipitation.

## 5. Conclusions

The description of the female *D. tejensis* presented here allows for its identification that was previously impossible to even guess based on the description of the male. This is also important because *D. tejensis*, although limited in its range, is not as rare as its recent discovery might suggest. This finding also made it possible to update the only existing, nearly 100-year-old key to all Nearctic species of *Dielis*, although its current version is still not final. Future studies should clarify, among others, the taxonomic status of the *D. plumipes* subspecies and the generic affiliation of *D. pilipes*. Of interest, some phenotypic traits in *D. tejensis*, such as the yellow pattern of the integument, turned out to be partly season-dependent, a phenomenon that was previously unknown in Scoliidae and was rarely observed in other Hymenoptera. It draws attention to the fact that unrecognized seasonal phenotypic changes may complicate interpretation of random genetic variation, trophic forms and even higher-ranking taxa. Lastly, the finding of close overlap between the ranges of *D. tejensis* and one of the main food-plants for its imagines, *H. artemisiifolius*, remains puzzling and may suggest the existence of a trophic relationship between this plant and still-unknown beetle host of *D. tejensis* larvae or the development of specific yet similar environmental requirements by *D. tejensis* and *H. artemisiifolius*.

## Data Availability

The original contributions presented in this study are included in the article. Further inquiries can be directed to the corresponding author.

## Supplementary Materials

The following supporting information can be downloaded at https://www.mdpi.com/article/10.3390/insects17030295/s1, Table S1: The geographic coordinates of the *D. tejensis* and *D. plumipes fossulana* locations shown in Figure 4; Table S2: The list of authors of the *D. tejensis* and *D. plumipes fossulana* records in iNaturalist used in Figure 4; Figure S1: Preliminary assessment of the seasonality of the *D. tejensis* population in the Brazos Valley (Burleson Co., Texas, at locations crossing the study area over a distance of 10 km).
