# Supplementary material for: Alloreferent and Apparent Seasonal Polyphenism of Dielis tejensis with an Updated Key to Nearctic Dielis Species (Hymenoptera: Scoliidae)
Source: Insects. 2026 Mar 9;17(3):295. doi: 10.3390/insects17030295 (PMC13027304; doi:10.3390/insects17030295)
Supplement: Supplementary file 1 [file insects-17-00295-s001.zip › Figure S1.pdf]

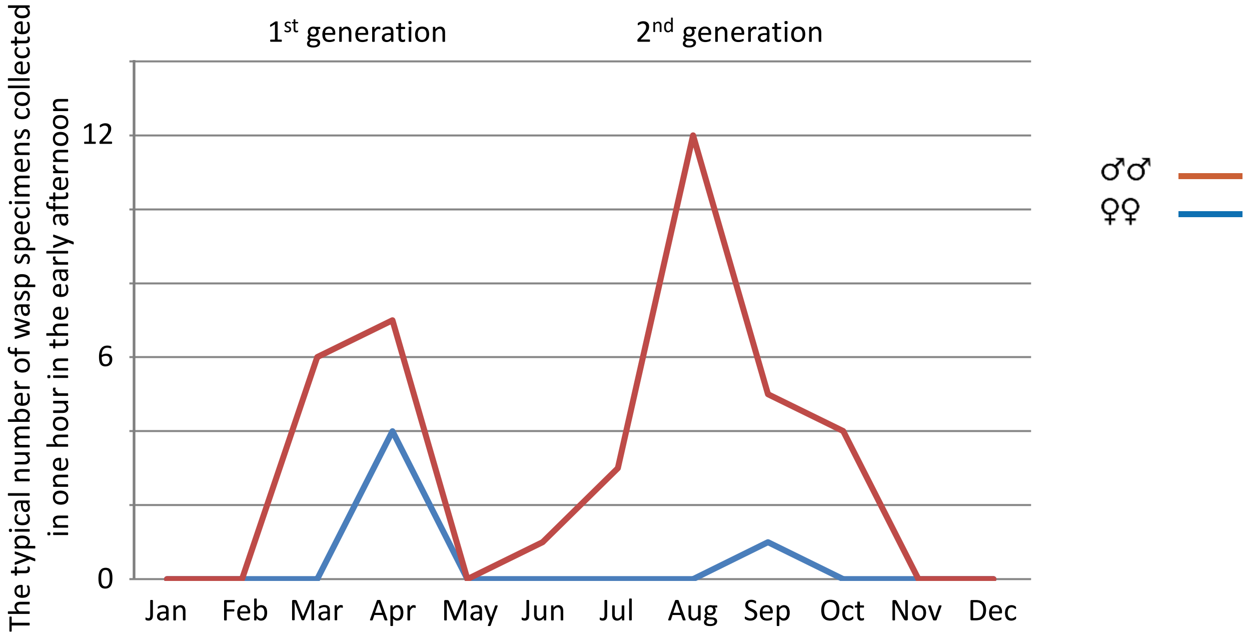

Figure S1. Preliminary assessment of the seasonality of the *D. tejensis* population in the Brazos Valley (Burleson Co., Texas, at locations crossing the study area over a distance of 10 km).
